# Effect of *Epichloë* Endophytes on Growth of Grass Pathogenic Fungi

**DOI:** 10.3390/microorganisms14030648

**Published:** 2026-03-13

**Authors:** Cuiling Wan, Xiuzhang Li, Qian Shi

**Affiliations:** 1 State Key Laboratory of Plateau Ecology and Agriculture, Qinghai University, Xining 810016, China; wancuiling2002@163.com (C.W.); xiuzhang@qhu.edu.cn (X.L.); 2 Qinghai Academy of Animal and Veterinary Science, Qinghai University, Xining 810016, China

**Keywords:** *Achnatherum inebrians*, endophytic fungi, *Festuca sinensis*, *Hordeum brevisubulatum*, interaction, antifungal activity

## Abstract

Endophytic fungi widely colonize plant tissues without causing severe disease, protect hosts from pathogenic microorganisms, and represent a key potential resource for novel biocontrol agents. To explore the biocontrol potential of endophytic *Epichloë* fungi and their correlation with alkaloids, 12 *Epichloë* strains were isolated from six different geographic sites of *Festuca sinensis*, *Achnatherum inebrians*, and *Hordeum brevisubulatum*. The antifungal activity of these strains was evaluated against five phytopathogenic fungi (*Alternaria alternata*, *Bipolaris sorokiniana*, *Curvularia lunata*, *Fusarium avenaceum*, and *Drechslera erythrospila*) using dual-culture assays, which measured the inhibition of both colony growth and spore germination. Concurrently, the concentrations of ergonovine and ergine were quantified in the liquid cultures of each *Epichloë* strain. The results showed that 12 *Epichloë* strains had varying degrees of inhibitory effects on the colony growth and spore germination of five pathogenic fungi. Among these, strain F2 had the highest inhibition rate on the spore germination of *B. sorokiniana* (87.73%), while strain H3 had the lowest inhibition rate on *F. avenaceum* (7.89%). The concentrations of ergonovine and ergine were positively correlated with the inhibition rate of pathogenic fungi, but the degree of these correlations varied among different pathogenic fungi. This study provides further evidence for *Epichloë*-mediated pathogen inhibition and establishes a basis for their development as biocontrol agents in agricultural systems.

## 1. Introduction

Grasses are threatened by numerous phytopathogenic microorganisms, which cause substantial damage to host plants [1]. A wide variety of pathogens can infect grasses, including fungi, bacteria, viruses, phytoplasmas, viroids, and nematodes. Among these, fungal pathogens account for more than 80% of total plant diseases [2]. Although considerable progress has been made in understanding fungal pathogenicity and developing control strategies over the past century, plant fungal diseases still represent a major constraint in agricultural production [3]. Plant diseases are the main factors affecting the health of natural grasslands. They not only reduce the yield and quality of forage grasses, but also restrict the sustainable utilization of grasslands and the development of animal husbandry [4]. By applying chemical pesticides, the losses caused by diseases can be mitigated to a certain extent. However, chemical pesticides not only cause environmental problems but also select for strains with resistance [5,6]. Therefore, applying beneficial endophytic microorganisms as biocontrol agents for disease management is an economical and effective strategy [7]. In pastoral, turfgrass, and natural grassland ecosystems, many cool-season grasses form symbiotic associations with endophytic fungi of the genus *Epichloë*. These endophytes are well known for their ability to produce antimicrobial metabolites, which effectively inhibit the growth of pathogens both in vitro and in planta [1,8,9,10].

Grass endophytes, comprising both sexual (*Epichloë* spp.) and asexual (*Neotyphodium* spp.) clavicipitaceous fungi (Clavicipitaceae, Hypocreales, Ascomycota), form symbiotic associations with some cool-season grasses (family *Poaceae* subfamily *Pooideae*) [11]. Nowadays, the *Neotyphodium* and *Epichloë* genera are commonly referred to collectively as *Epichloë* endophytes [12]. Endophytic fungi often exhibit host specificity, with each species typically colonizing only one or a few grass hosts [13]. These endophytes grow systemically in the aboveground plant parts, obtaining nutrients from the apoplast of developing leaves and stems near the shoot meristems [14]. In addition, they can be vertically transmitted via host seeds [15]. There is growing research interest in these fungal endophytes due to their ability to confer multiple benefits on host plants. These benefits include enhanced growth and increased tolerance to both abiotic stresses (e.g., drought, salt, temperature, and heavy metals) [16,17,18,19,20] and biotic stresses (e.g., herbivores and nematodes) [21]. The long-term co-evolution and stable symbiotic relationship between grasses and *Epichloë* fungi imply that these endophytes play an important role in plant–pathogen interactions [22]. Studies have shown that *Epichloë* endophytes can inhibit the growth and pathogenicity of various pathogenic fungi through multiple in vitro and in planta mechanisms [23]. However, this effect varies depending on the specific pathogenic fungus and endophyte strain. Although alkaloids have been hypothesized to play a role in plant–pathogen interactions [24], direct evidence for an association between alkaloid levels and pathogen suppression remains insufficient, and the underlying mechanisms warrant further investigation.

*Achnatherum inebrians* is one of the most toxic wild grasses in the northern natural grasslands of China, yet as a plant adapted to saline habitats in arid and semi-arid regions, it holds potential for ecological restoration and soil conservation [25]. *Festuca sinensis* and *Hordeum brevisubulatum* are important high-quality forage grasses in China. *F. sinensis* contributes to the restoration of degraded alpine meadows through soil conservation, while *H. brevisubulatum* shows promise in the improvement of saline–alkaline land; both grasses possess high forage value and play vital roles in maintaining regional ecological stability [26,27]. All three have a relatively high rate of *Epichloë* endophytic fungal infection in the natural grasslands [28,29,30,31]. The *A. inebrians* endophyte symbiotic system has been extensively studied in China’s grass endophyte research. Researchers have conducted comprehensive investigations into its physiological, biological, and chemical aspects [24,29,32,33]. Studies have shown that *Epichloë* can significantly enhance drought tolerance and powdery mildew resistance in *A. inebrians* [34,35]. The biological and ecological characteristics of the *F. sinensis-Epichloë* endophyte symbiont have also been characterized [28]. Similarly, the *Epichloë* symbiont improves seed germination and cold resistance in *F. sinensis* [17,19,36,37]. For *H. brevisubulatum*, endophyte infection significantly enhances host salt tolerance and waterlogging tolerance [38,39], while also improving nutrient uptake under salt stress [40]. Furthermore, studies have shown that the presence of *Epichloë* endophytes in *H. brevisubulatum* increases acetone content. This increase in acetone may significantly enhance antifungal activity against *Alternaria alternata*, *Bipolaris sorokiniana*, *Fusarium avenaceum*, and *Trichoderma viride* [41].

Evidence indicates that *Epichloë* endophytes can directly inhibit the growth of plant-pathogenic fungi [42,43,44], thereby enhancing host resistance [45,46,47,48]. To extend these findings and assess the potential role of specific alkaloids, it was hypothesized that geographically diverse *Epichloë* strains exhibit variable but direct antifungal activity in vitro, and further, that this activity is positively correlated with their concentration of the ergot alkaloids ergonovine and ergine. To test this hypothesis, in vitro dual-culture assays were conducted using 12 *Epichloë* strains isolated from three grass species (*F. sinensis*, *A. inebrians*, and *H. brevisubulatum*) across six sites, against five grass pathogenic fungi (*Alternaria alternata*, *Bipolaris sorokiniana*, *Curvularia lunata*, *Fusarium avenaceum*, and *Drechslera erythrospila*). The inhibitory effects on both colony growth and spore germination were measured, and the concentrations of ergonovine and ergine in liquid cultures were measured to analyze their correlation with antifungal activity. This work adds to the growing evidence of *Epichloë*-mediated pathogen inhibition and lays the groundwork for evaluating their potential as biocontrol agents in agricultural development.

## 2. Materials and Methods

### 2.1. Biological Materials

Endophytic fungi were isolated from *F. sinensis*, *A. inebrians*, and *H. brevisubulatum* with high infection rates. Plant samples were collected from six different sites, and two strains were selected from each site as experimental backups. The specific sampling locations, together with their latitude, longitude, and altitude, are listed in Table 1. The collected single-plant samples were examined under a microscope using the aniline blue staining method of stem pith [49] to determine whether they carried endophytic fungi. The stems of the plants detected with endophytic fungi were cut into small segments over 1 cm in length and then surface sterilized (75% absolute ethanol for 2 min, 1% sodium hypochlorite for 3 min, and rinsed with sterile water three times). After removing surface moisture with sterile filter paper, the stem segments were inoculated onto antibiotic potato dextrose agar (ABPDA) medium and incubated in the dark at 25 °C. Five fungal pathogens were used in this study. *B. sorokiniana*, *F. avenaceum*, and *A. alternata* were isolated from *A*. *inebrians*, while *D*. *erythrospila* and *C*. *lunata* were isolated from *Lolium perenne*.

The *F. sinensis* samples were collected from Xiahe County, Gansu Province, and Guide County, Qinghai Province, China; the isolated *Epichloë* strains were designated F1, F2, F3, and F4. The *H. brevisubulatum* samples were collected from Linze County and Ganzhou District, Gansu Province, China; the isolated *Epichloë* strains were designated H1, H2, H3, and H4. The *A. inebrians* samples were collected from Sunan Yugur Autonomous County and Hezuo City, Gansu Province, China; the isolated *Epichloë* strains were designated A1, A2, A3, and A4 (Table 1).

### 2.2. The Inhibition Rate of Grass Pathogenic Fungi by Epichloë Endophytes In Vitro

A mycelial plug (4 mm diameter) of each endophytic fungus was taken from the margins of actively growing colonies and transferred face down in the centre of a 90 mm diameter Petri dish of ABPDA. After the cultures of *Epichloë* endophytes were grown on ABPDA at 22 ± 1 °C in the dark for four weeks, they were inoculated with grass pathogenic fungi. For each treatment, three biological replicates (endophyte-inoculated dishes) were prepared. For each biological replicate, three mycelial plugs (4 mm in diameter) from a one-week-old culture of the fungal pathogen were evenly spaced and inoculated on the dish, serving as technical replicates. Meanwhile, five different pathogenic fungal colonies (4 mm in diameter) were evenly spaced and inoculated on the edge of a blank ABPDA medium as a blank control. All dishes were sealed with laboratory film (Parafilm, Pechiney Plastic Packaging, Menasha, WI, USA), then put the dish at 22 ± 1 °C in the dark for one week. After one week of observing the growth condition of pathogenic fungi, we measured the pathogenic fungal colony diameter and the distance between the endophytic fungi and pathogenic fungi and calculated the inhibition rate using the formula:

(1) Ir (%) = [(Dck − Dpa)/Dck] × 100,

Ir is inhibition rate and Dck is the mean colony diameter of the pathogenic fungus in the control group. Dpa is the mean colony diameter of the pathogenic fungus in the endophyte-treated group.

### 2.3. Effect of the Culture Supernatant of Endophytic Fungi on Pathogenic Fungi Colony Growth

ABPDA medium (150 mL) was dispensed into a 250 mL Erlenmeyer flask and sterilized. Following sterilization and cooling, two 4 mm mycelial plugs of the endophytic fungus were added aseptically. After sealing with Parafilm, the flasks were incubated in the dark at 22 ± 1 °C with agitation at 140 rpm on an orbital shaker. After four weeks, we transferred the endophytic fungi liquid culture to a 50 mL centrifuge tube and centrifuged it at 3500 rpm. After centrifugation, we took the supernatant and stored it in a 4 °C refrigerator for later use.

Sterilized 90 mm Petri dishes were individually supplemented with 1 mL of the endophytic fungal culture and 20 mL of melted, sterile ABPDA medium. After the medium solidified, a pathogenic fungal mycelial plug (4 mm in diameter) was inoculated in the center of each prepared plate. For each treatment, three biological replicates were prepared, each consisting of three technical replicates (plates). As a blank control, another set of sterilized 90 mm Petri dishes was prepared by adding 1 mL of sterile liquid PDA medium and 20 mL of melted, sterile ABPDA medium. Five different pathogenic fungi were then individually inoculated onto these control plates. All plates were sealed with laboratory film (Parafilm, Bemis Flexible Packaging, Neenah, WI, USA) and incubated in the dark at 22 ± 1 °C for one week. After a week, we measured the colony diameter and calculated the inhibition rate. The formula for calculating the inhibition rate is the same as Formula (1).

### 2.4. Effects of Epichloë Endophytes on Spore Germination of Grass Pathogenic Fungi

Under aseptic conditions, 5 mL of sterile water was added to each plate colonized by the pathogenic fungus. The mycelial mat was then scraped off using a sterile spreader and filtered through sterile gauze into a pre-prepared Erlenmeyer flask. The resulting suspension was homogenized thoroughly using sterile inoculation loops to ensure complete spore dispersion. A small aliquot was taken using a rubber-tipped dropper to prepare a wet mount for microscopic observation. The spore concentration was adjusted with sterile water until the spore density reached no less than 100 spores per field of view under 400× magnification.

For the experimental treatment, 1 mL of endophytic fungal culture supernatant and 1 mL of pathogenic fungal spore suspension were mixed on a sterile double-concave slide and covered with a coverslip to prepare a wet mount. For the blank control (CK), 1 mL of sterile liquid ABPDA medium was mixed with 1 mL of the same pathogenic fungal spore suspension on a separate slide. Each mixture was gently stirred with a dissecting needle to ensure uniformity. The spore concentration was examined under a microscope at 400× magnification and adjusted to 80–100 spores per field of view. The prepared slides were placed in 11 cm diameter Petri dishes under humid conditions and incubated at 22 ± 1 °C. Spore germination of the grass pathogens was examined every 2 h until the germination rate in the blank control group exceeded 65%. For each treatment, three biological replicates were examined, with three fields of view counted per replicate as technical replicates, and germinated spores were counted using a hemocytometer. The inhibition rate was calculated using the following formula:

(2) Ir′ (%) = [(Nck − Npa)/Nck] × 100,

Ir’ is the inhibition rate, Nck is the mean number of germinated spores in the control group, and Npa is the mean number of germinated spores in the treatment group.

### 2.5. Determination of Ergot Alkaloids

The concentrations of ergonovine and ergine were determined by HPLC analysis following a modified method adapted from Zhang et al. [50] and Liu [51]. Briefly, 500 μL of endophytic fungal culture supernatant was mixed with 1 mL of 20% acetic acid in a 2 mL centrifuge tube. The mixture was sonicated for 5 min, vortexed for 2 min, and then centrifuged at 1000× *g* for 5 min. A 0.5 mL aliquot of the resulting supernatant was loaded onto a PCX column pre-activated with 2 mL of methanol. The column was sequentially rinsed with 2 mL of purified water and eluted with 1 mL of a 95% methanol solution containing 5% ammonia. The eluate was collected, and a 0.25 mL portion was passed through a 0.22 μm organic phase filter into a 1.5 mL amber vial. All prepared samples were stored in the dark at 4 °C until analysis. HPLC analysis was performed on an Agilent 1100 system equipped with an Agilent C18 column (250 mm × 4.6 mm, 5 μm particle size). The mobile phase consisted of (A) 0.1 mol·L^−1^ ammonium acetate and (B) acetonitrile:0.1 mol·L^−1^ ammonium acetate (3:1, *v*/*v*), delivered at a flow rate of 1.0 mL·min^−1^. The elution gradient was programmed as follows: 95% A (0–10 min), linearly decreased to 80% A (10–20 min), further decreased to 50% A (20–30 min), and finally returned to 95% A (30–35 min). Detection was carried out at 312 nm. Alkaloid quantification was achieved by comparing peak areas of 20 μL injections with external standard calibration curves.

### 2.6. Statistical Analysis

Data are presented as the mean ± standard error of three replications. The normality of the data was tested using the Shapiro–Wilk test, and the data conformed to a normal distribution. Then, the experimental data were statistically analyzed using one-way analysis of variance (ANOVA), followed by Duncan’s post hoc test at the 0.05 level, with SPSS statistical software (Version 27.0, Chicago, IL, USA). An RDA (redundancy analysis) was conducted using the Canoco software program (Version 5.02 trial, Windows release) to analyze the relationship between the concentration of ergonovine and ergine alkaloids in liquid culture medium by *Epichloë* endophytes and the inhibition rate of the fungal pathogen. All data were centered and standardized prior to analysis. The significance of the RDA axes was tested using Monte Carlo permutation tests as implemented in the software, and the overall model was significant.

## 3. Results

### 3.1. Effect of the Colony Growth of Grass Pathogenic Fungi in the Culture Medium Inoculated by Epichloë Endophytes

Compared with the controls, all strains of *Epichloë* endophytes significantly (*p* < 0.05) inhibited the colony growth of *D. erythrospila*, *B. sorokiniana*, *C. lunata*, *F. avenaceum*, and *A. alternata* (Table 2). Strain A1, isolated from *A. inebrians*, exhibited the highest inhibition rate against *B. sorokiniana* (73.24%), while strain H2 from *H. brevisubulatum* showed the lowest inhibition rate against *F. avenaceum* (8.41%).

All five fungal pathogens were inhibited to varying degrees by the *Epichloë* endophytes, with significant differences in inhibitory effects among endophytic strains against the same pathogen (*p* < 0.05). Notably, strain A1 exhibited significantly stronger inhibition than the other tested strains against most pathogens, particularly *D. erythrospila*, *B. sorokiniana*, *C. lunata* and *F. avenaceum*. In contrast, strain H3 showed the highest inhibitory activity specifically against *A. alternata*.

The inhibitory effect of a given *Epichloë* strain also varied significantly across different pathogens (*p* < 0.05). Overall, *B. sorokiniana* was the most sensitive pathogen, with the majority of endophytic strains exerting strong inhibitory effects against it. In contrast, the other four pathogens displayed strain-specific susceptibility: *F. avenaceum*, *C. lunata*, *A. alternata* and *D. erythrospila* were most strongly inhibited by strains A1, H1, H3 and H4, respectively.

### 3.2. Effect of the Growth of Grass Pathogenic Fungi Under Liquid Culture Medium by Epichloë Endophytes

#### 3.2.1. Effect of the Colony Growth of Grass Pathogenic Fungi Under Liquid Culture Medium by *Epichloë* Endophytes

Compared with the controls, all liquid culture media of *Epichloë* endophytes significantly (*p* < 0.05) inhibited the colony growth of *D. erythrospila*, *B. sorokiniana*, *C. lunata*, *F. avenaceum*, and *A. alternata* (Table 3). Strain A1, isolated from *A. inebrians*, showed the most significant inhibitory effect on the colony growth of *B. sorokiniana*, with an inhibition rate of 74.68%. In contrast, strain H2 from *H. brevisubulatum* exhibited the weakest inhibition against *F. avenaceum* (9.83%).

All five fungal pathogens were inhibited to varying degrees by the liquid culture medium of *Epichloë* endophytes, and significant differences in antifungal activity were detected among endophytic strains (*p* < 0.05). Overall, strain A1 displayed significantly stronger inhibitory effects than most other strains against *D. erythrospila*, *B. sorokiniana*, and *C. lunata*. Strain A2 also showed high inhibitory activity against *F. avenaceum*, while strain F2 exhibited strong inhibition against *D. erythrospila* and *A. alternata*. In addition, strain F4 was among the most effective isolates against *A. alternata*.

The inhibitory effects of the same *Epichloë* endophytic strain varied significantly across different grass pathogenic fungi. Strains F1, F2, F3, F4, H1, H2, H3, and H4 demonstrated significantly higher inhibition rates against *D. erythrospila* than against the other four pathogenic fungi (*p* < 0.05). Meanwhile, strains A1, A2, A3, and A4 showed the most pronounced inhibition against *B. sorokiniana*.

#### 3.2.2. Effect of the Fungal Pathogen Spore Germination Under Liquid Culture Medium by *Epichloë* Endophytes

Compared with the controls, all liquid culture media of *Epichloë* endophytes significantly (*p* < 0.05) inhibited spore germination of *D. erythrospila*, *B. sorokiniana*, *C. lunata*, *F. avenaceum*, and *A. alternata* (Table 4). Strain F2, isolated from *F. sinensis*, exhibited the highest inhibition rate against *B. sorokiniana* spore germination (87.73%), whereas strain H3 from *H. brevisubulatum* showed the weakest inhibition against *F. avenaceum* (7.89%).

All five fungal pathogens showed varying degrees of spore germination inhibition by *Epichloë* endophytes. Significant differences in inhibitory effects were observed among different endophytic strains against the same pathogen (*p* < 0.05). Overall, strain F3 exhibited the strongest inhibitory effect on *D. erythrospila* spore germination, outperforming other tested strains. For *B. sorokiniana* and *C. lunata*, strain F2 was the most effective inhibitor. Meanwhile, strain A3 showed the highest inhibitory activity against *F. avenaceum*. In addition, strains F2 and F3 displayed significantly stronger inhibition of *A. alternata* spore germination. Their inhibitory effects were notably higher than those of other strains.

The inhibitory effects of the same *Epichloë* endophytic strain varied significantly across different grass pathogenic fungi. All 12 strains exhibited the strongest inhibitory effect on the spore germination of *B. sorokiniana*, while showing the weakest inhibition against *F. avenaceum*.

#### 3.2.3. Relationship Between the Concentration of Ergonovine and Ergine Alkaloids in Liquid Culture Medium by *Epichloë* Endophytes and the Inhibition Rate of the Fungal Pathogen

**Figure 1:**
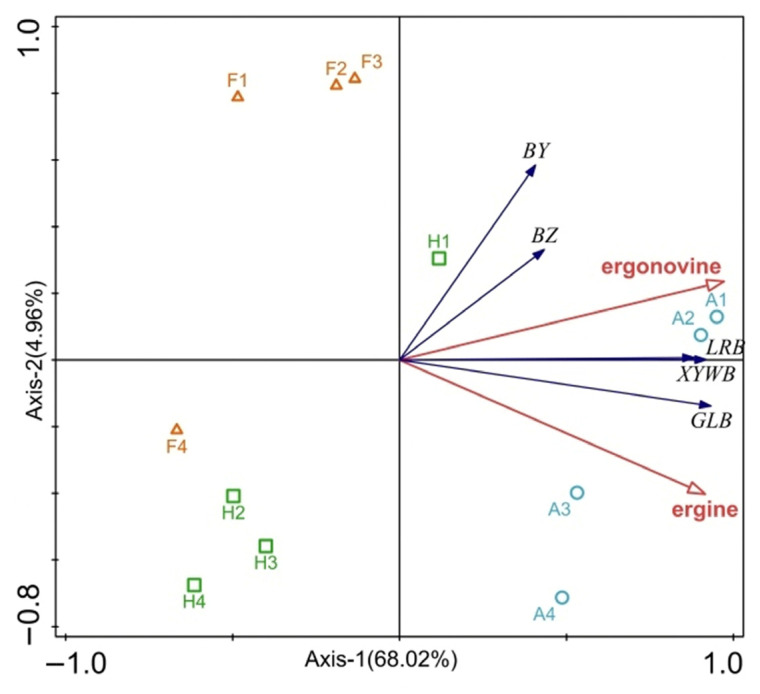
The result of RDA analysis among the concentration of alkaloids in liquid culture medium by *Epichloë* endophytes and the inhibition rate of the fungal pathogen. Note: BY indicated *Drechslera erythrospila*; BZ indicated *Alternaria alternata*; XYWB indicated *Curvularia lunata*; LRB indicated *Bipolaris sorokiniana*; GLB indicated *Fusarium avenaceum*.

The first two RDA axes explained 68.02% and 4.96% of the total variance, respectively, cumulatively accounting for 72.98% of the total variation (Figure 1). The concentrations of two ergot alkaloids (ergonovine and ergine) in the liquid culture medium of *Epichloë* endophytes showed a positive correlation with the inhibition rates against five pathogenic fungi (*D. erythrospila*, *A. alternata*, *C. lunata*, *B. sorokiniana*, and *F. avenaceum*), but their inhibitory effects varied among different pathogens. Specifically, compared to ergine, ergonovine exhibited a stronger correlation with the inhibition rates of *D. erythrospila* and *A. alternata*, and its correlation with the inhibition rates of *B. sorokiniana* and *C. lunata* was also significantly stronger than that of ergine. In contrast, ergine showed a stronger correlation with the inhibition rate of *F. avenaceum*. Among the endophytic fungal strains, strains A1 and A2 had the highest ergonovine concentration and simultaneously displayed the strongest inhibitory effects on *B. sorokiniana* and *C. lunata*. In contrast, strains F4, H2, H3, and H4 had relatively low concentrations of both alkaloids and generally weak inhibitory activity against all five pathogenic fungi.

## 4. Discussion

The mechanisms by which endophytic fungi enhance disease resistance in host grasses may involve the direct inhibition of spore germination [43,52], the enhancement of host plant resistance, and the production of antifungal compounds by the *Epichloë* endophytic symbiont [53,54]. Alternatively, endophytic infection leads to the formation of fungal mycelial networks or hyphal sheaths within host plant tissues. These structures occupy specific ecological niches in grass tissues and may trigger rejection responses from pathogenic fungi [55]. The antifungal activities of 12 *Epichloë* strains isolated from three grass species were evaluated against five pathogenic fungi using dual culture and liquid culture assays, with impacts on colony growth and spore germination assessed. Consistent with the hypothesis proposed in Section 1, all 12 *Epichloë* endophytes exhibited varying degrees of inhibition on both colony growth and spore germination of the pathogenic fungi. A significant positive correlation was observed between the inhibition rates against the five pathogenic fungi and the alkaloid concentrations in the liquid culture media of the different *Epichloë* endophyte strains. Different *Epichloë* strains exhibit varying alkaloid concentrations in the liquid culture medium, with corresponding differences in their inhibitory effects on pathogenic fungi.

This study indicates that strains A1 and A2 produced the highest ergonovine concentrations and simultaneously showed the strongest inhibitory effects against *B. sorokiniana* and *C. lunata*. This is consistent with the previous research by Sun et al. [24], who reported the presence of ergonovine and ergine in endophyte-infected *A. inebrians*, while these alkaloids were absent in endophyte-free plants. Their study also demonstrated that extracts from endophyte-infected plants exhibited higher inhibition of pathogenic fungal growth, and that alkaloid concentration was positively correlated with the inhibition of spore germination. Therefore, ergonovine and ergine alkaloids produced by *Epichloë* endophytes may contribute to the enhancement of disease resistance observed in their host grasses. It is important to note, however, that the inhibitory effect of endophytic fungi on plant pathogenic fungi is not solely dependent on alkaloids. Various non-alkaloid secondary metabolites have also been implicated, including sesquiterpenes [54], phenolic glycerides [56], hydroxyl unsaturated fatty acids [57], aromatic sterols [58], indole derivatives (indole-3-acetic acid (IAA) and indole-3-ethanol), diacetamides [54], as well as volatile compounds such as Chokol K and methyl esters [59]. Different *Epichloë* species associated with different grass hosts produce distinct metabolite profiles, which in turn lead to varying inhibitory effects on pathogenic fungal growth [60].

The present study revealed that *Epichloë* strains derived from different host grass species differed in their inhibitory effects on pathogen colony growth and spore germination. In terms of spore germination inhibition, strains isolated from *F. sinensis* generally exhibited stronger antifungal activity than those from *A. inebrians* and *H. brevisubulatum*. This host-dependent variation in inhibitory activity is consistent with the findings of Xie et al. [61], who also reported that the antifungal potential of *Epichloë* endophytes differed significantly according to their original host species. Furthermore, Yang et al. [41] compared the antifungal activity of acetone extracts from endophyte-infected and endophyte-free host grasses against four fungi: *A. alternata*, *B. sorokiniana*, *F. avenaceum* and *Trichoderma viride*. They found that extracts from infected plants showed significantly stronger inhibition at all tested concentrations (*p* < 0.05), and markedly suppressed mycelial growth, conidial germination, and germ tube elongation. These results clearly demonstrated that endophytic infection significantly enhances the antifungal activity of host grass acetone extracts. Previous studies have indicated that the inhibitory effects of endophytic fungi on pathogens are influenced by endophyte genotype [60], which is consistent with the results of the present study.

Different *Epichloë* strains isolated from different geographic sites of the same species showed significant differences in their inhibitory rates of pathogenic fungi. The inhibition rates of colony growth of *D. erythrospila* and *C. lunata* by the liquid culture media of strains A1 and A2, isolated from *A. inebrians* in Sunan, were significantly different (*p* < 0.05) compared with those of strains A3 and A4, isolated from *A. inebrians* in Hezuo. These two sampling sites are approximately 510 km apart. Furthermore, such inhibitory variation also exists among different *Epichloë* strains. Similarly, Liu (2019) observed that strain XH from Xiahe and strain PA from Ping’an, both isolated from *F. sinensis*, displayed significantly distinct inhibitory activity against *A. alternata* [51]. This phenomenon may be attributed to the diverse environmental pressures that shape the host–endophyte symbiosis. Distinct habitats can modulate the physiological and metabolic profiles of *Epichloë* endophytes, thereby altering the production, composition, and yield of antifungal metabolites synthesized by the symbiotic system. These metabolic differences ultimately lead to the observed variation in inhibitory activity against pathogenic fungi [62]. However, further in-depth studies are needed to understand the specific inhibiting molecules.

There are some differences in the inhibition activity of pathogenic fungi between different strains of *Epichloë* endophytes isolated from the same geographic sites of the same species of plant. For example, strain H1 inhibited the colony growth of the five pathogenic fungi in the following order of decreasing inhibition rates: *C. lunata*, *B*. *sorokiniana*, *A*. *alternata, D. erythrospila*, and *F. avenaceum*, but the order for strain H2 was *B. sorokiniana*, *C. lunata*, *D. erythrospila, A. alternata*, and *F. avenaceum*. This result is similar to Li’s findings on the interaction between *E. gansuensis* and pathogens [46]. In their study, when tested against four pathogenic fungi, strain L112 inhibited colony growth in the following order of decreasing sensitivity: *B. sorokiniana*, *C. lunata*, *F. avenaceum* and *A. alternata*, but the order for L113 was *B. sorokiniana*, *F. avenaceum*, *A. alternata* and *C. lunata*. L112 and L113 are *E. gansuensis,* which were isolated from the same geographic populations of *A. inebrians*, but their inhibitory effects on pathogenic fungi are significantly different. Niones and Takemoto also reached similar results. Although both E364 and E365 were isolated from *Festuca longifolia* in Switzerland, only E364 exhibited inhibitory effects against *C. graminicola* [63]. This differential activity is likely attributable to underlying genetic heterogeneity between the isolates.

The results of this study also showed that endophytic fungi exhibit varying inhibitory effects on pathogenic fungi under different culture conditions. Taking strain F4 as an example, its inhibitory activity against the five tested pathogenic fungi differed between solid and liquid culture media. Such differences in inhibitory activity under different culture conditions were also observed in other endophyte strains. For instance, Ma and Nan [2] reported that strain N-A1 isolated from *L. perenne* also showed distinct inhibitory effects on pathogen colony growth between solid and liquid culture assays. This condition-dependent variation in inhibitory activity is likely due to different inhibitory mechanisms under distinct culture conditions [42]. Previous studies have found that the main mechanism is hyperparasite or niche competitive when the colony of *Epichloë* endophytes and pathogenic fungi has a direct action on the culture medium. The *Epichloë* endophytes invade pathogen hyphae and enzymatically hydrolyze the pathogenic fungus cells [61], or compete against pathogenic fungus nutrients and niche space, and achieve antagonism between pathogenic fungi [30]. In liquid culture, however, *Epichloë* endophytes mainly inhibit pathogenic fungi by secreting active compounds such as indole derivatives, sesquiterpenes, and alkaloids [54,64,65]. Furthermore, whether different culture media and conditions affect the types and quantities of antifungal metabolites produced by *Epichloë* endophytes requires further investigation.

The results indicate that all 12 strains exhibited significant inhibition of pathogenic fungal growth. This finding, however, contrasts with that of Li et al., who reported that both *N. lolii* and *N. coenophialum* promoted the growth of *C. lunata* and *A. alternata*, and that strains NgL121, NgL141, NgL232, and NgS1a1 enhanced the sporulation of *Fusarium acuminatum* [46]. Generally, the variations in the inhibitory effects of endophytic fungi result from the combined action of multiple factors, including pathogen strains, grass genotypes or varieties, growth conditions, endophyte status, and so on [42,44,66]. Although some endophytes produced antifungal substances in vitro [8], they may not be able to produce these compounds in sufficient quantities in the grass to protect it from fungal diseases [52,56]. Alternatively, the inhibitory substances produced in vitro may differ from those synthesized in planta [42]. After all, external culture conditions in vitro are quite different from the native growth conditions of endophyte fungi. Furthermore, regarding secondary metabolites, which are crucial for symbiotic protection, the concentrations produced by the endophyte alone are far lower than those of compounds such as alkaloids generated by endophyte–grass symbiosis [60]. Therefore, future research should focus on identifying the specific antagonistic compounds produced by these *Epichloë* endophytes and elucidating their mechanisms of action. Such efforts will support the potential of *Epichloë* endophytes as promising biocontrol agents for the sustainable management of grass fungal diseases.

## Figures and Tables

**Table 1:** Information on strain codes, host plants, and collection sites of *Epichloë* endophytic fungi.

Host	*Epichloë* spp.	Region	Elevation	Longitude and Latitude
*Festuca sinensis*	F1	Xiahe	3047 m	35°07′ N, 102°26′ E
	F2			
	F3	Guide	3565 m	36°21′ N, 101°27′ E
	F4			
*Hordeum brevisubulatum*	H1	Ganzhou	1473 m	38°57′ N, 100°25′ E
	H2			
	H3	Linze	1450 m	37°29′ N, 102°54′ E
	H4			
*Achnatherum inebrians*	A1	Sunan	2663 m	38°55′ N, 99°39′ E
	A2			
	A3	Hezuo	2735 m	35°06′ N, 102°51′ E
	A4			

**Table 2:** The inhibitory rate of endophytic fungi on grass-pathogenic colony growth. Different lowercase letters within the same column indicate significant differences in the inhibitory rates of different endophytic fungal strains against the same pathogenic fungi (*p* < 0.05). Different uppercase letters within the same row indicate significant differences in the inhibitory rates of the same strain against different pathogenic fungi (*p* < 0.05).

	*D. erythrospila*	*B. sorokiniana*	*C. lunata*	*F. avenaceum*	*A. alternata*
F1	38.13 ± 4.38 BC fghij	44.28 ± 1.54 A jk	39.27 ± 2.87 B jk	9.20 ± 2.36 E ghij	37.26 ± 0.95 BCD gh
F2	39.03 ± 3.86 CD defghi	50.42 ± 0.64 A gh	42.63 ± 3.97 BC efghij	12.80 ± 1.99 E g	45.53 ± 1.82 B bcd
F3	45.11 ± 2.33 BCD bcde	56.00 ± 4.11 A ef	45.32 ± 2.41 BC efghi	22.48 ± 0.65 E e	48.37 ± 6.04 AB ab
F4	44.29 ± 5.54 BCD bcdef	61.79 ± 8.62 A bcde	48.64 ± 4.58 AB cdef	18.94 ± 3.02 E ef	44.69 ± 3.60 BC bcde
H1	42.24 ± 6.8 ABCD bcdefg	47.04 ± 1.77 AB ij	47.20 ± 2.56 A cdefgh	10.30 ± 1.98 E gh	46.97 ± 3.16 ABC abc
H2	47.45 ± 1.18 ABC bc	50.64 ± 3.25 A efg	50.22 ± 2.03 AB c	8.41 ± 1.64 E hijkl	27.63 ± 0.67 D l
H3	41.91 ± 3.15 D cdefgh	49.93 ± 1.66 AB ghi	48.67 ± 2.73 ABC cde	8.61 ± 3.60 E ghijk	50.57 ± 2.53 A a
H4	45.45 ± 4.35 A bcd	42.41 ± 2.43 AB kl	35.84 ± 1.34 D kl	9.63 ± 2.53 E ghi	42.38 ± 2.06 ABC bcdef
A1	52.87 ± 2.16 C a	73.24 ± 3.69 A a	66.09 ± 2.14 B a	52.25 ± 1.14 CD a	37.51 ± 0.53 E g
A2	48.66 ± 3.16 C ab	69.38 ± 3.65 A ab	65.17 ± 2.89 AB ab	48.09 ± 2.89 CD b	35.11 ± 1.95 E ghi
A3	35.11 ± 1.52 D ijkl	65.16 ± 1.18 A bc	49.88 ± 1.54 B cd	38.24 ± 1.11 C c	33.24 ± 1.37 DE ij
A4	36.26 ± 1.28 C ijk	64.53 ± 2.09 A bcd	48.56 ± 2.35 B cdefg	35.36 ± 0.43 CD d	31.49 ± 2.05 E ijk

**Table 3:** The inhibitory rate of the liquid culture medium of endophytic fungi on grass-pathogenic colony growth. Different lowercase letters within the same column indicate significant differences in the inhibitory rates of different endophytic fungal strains against the same pathogenic fungi (*p* < 0.05). Different uppercase letters within the same row indicate significant differences in the inhibitory rates of the same strain against different pathogenic fungi (*p* < 0.05).

	*D. erythrospila*	*B. sorokiniana*	*C. lunata*	*F. avenaceum*	*A. alternata*
F1	50.38 ± 1.03 A h	39.71 ± 2.80 B g	23.37 ± 1.41 CD j	10.26 ± 1.71 E hijk	26.72 ± 2.81 C h
F2	66.44 ± 1.44 A ab	39.60 ± 1.08 C gh	37.53 ± 4.22 CD e	14.57 ± 1.47 E fg	48.72 ± 5.51 B ab
F3	61.22 ± 1.39 A cde	50.20 ± 1.75 B e	34.81 ± 0.84 D ef	19.31 ± 0.93 E e	45.25 ± 1.74 C bcd
F4	57.93 ± 0.89 A fg	45.01 ± 1.57 C f	33.51 ± 3.05 D efg	15.52 ± 0.66 E f	49.57 ± 1.46 B a
H1	62.46 ± 5.36 A cd	33.74 ± 2.75 B i	27.76 ± 1.71 C h	11.29 ± 2.22 DE gh	12.90 ± 2.83 D jk
H2	60.74 ± 2.20 A cdef	27.72 ± 2.46 B jk	27.50 ± 1.41 BC hi	9.83 ± 2.16 DE hijkl	11.66 ± 1.42 D kl
H3	37.82 ± 2.53 A kl	30.84 ± 2.64 B ij	23.11 ± 3.25 C jk	10.62 ± 2.16 E hij	20.30 ± 3.66 CD hi
H4	41.04 ± 2.45 A k	27.16 ± 0.99 B kl	16.10 ± 2.28 C l	11.09 ± 1.67 E hi	15.78 ± 1.30 CD ij
A1	68.96 ± 1.82 B a	74.68 ± 2.39 A a	68.12 ± 1.96 BC a	61.44 ± 1.16 D ab	44.64 ± 2.34 E bcde
A2	63.61 ± 1.36 B c	70.62 ± 2.12 A ab	59.90 ± 1.64 D b	63.36 ± 1.14 BC a	46.94 ± 1.96 E abc
A3	48.90 ± 1.27 C hij	67.27 ± 0.96 A c	53.25 ± 1.34 B c	47.43 ± 1.06 CD c	40.37 ± 0.78 E f
A4	50.19 ± 1.15 BC hi	66.69 ± 0.85 A cd	52.04 ± 0.86 B cd	44.55 ± 1.11 D d	38.62 ± 1.15 E fg

**Table 4:** The inhibitory rate of the liquid culture medium of endophytic fungi on spore germination of grass-pathogenic. Different lowercase letters within the same column indicate significant differences in the inhibitory rates of different endophytic fungal strains against the same pathogenic fungi (*p* < 0.05). Different uppercase letters within the same row indicate significant differences in the inhibitory rates of the same strain against different pathogenic fungi (*p* < 0.05).

	*D. erythrospila*	*B. sorokiniana*	*C. lunata*	*F. avenaceum*	*A. alternata*
F1	63.00 ± 8.05 C ab	86.28 ± 2.60 A ab	53.41 ± 3.96 CD ghij	18.64 ± 3.99 E efgh	74.22 ± 2.44 B cd
F2	60.00 ± 8.19 D bcd	87.73 ± 2.01 A a	75.10 ± 3.29 C a	20.07 ± 4.36 E efg	82.81 ± 1.70 B a
F3	68.00 ± 1.80 D a	83.39 ± 1.30 A bc	74.30 ± 1.06 C ab	24.37 ± 1.29 E ab	80.86 ± 1.03 B ab
F4	55.50 ± 1.32 D def	79.78 ± 1.30 A def	71.08 ± 1.39 C abcd	21.15 ± 1.56 E de	75.00 ± 1.41 B c
H1	54.00 ± 2.18 BCD defg	70.40 ± 1.30 A j	56.63 ± 4.87 BC fghi	12.54 ± 3.53 E ghi	58.59 ± 3.34 B ef
H2	51.50 ± 3.04 CD fgh	80.87 ± 2.60 A cde	60.64 ± 3.14 B f	11.47 ± 3.12 E ij	51.95 ± 2.03 C gh
H3	37.50 ± 6.14 BCD ijkl	66.06 ± 7.46 A jk	45.38 ± 4.53 B jk	7.89 ± 2.58 E ijkl	45.31 ± 3.40 BC jk
H4	42.00 ± 4.09 B ijk	61.73 ± 4.86 A kl	38.15 ± 2.44 CD l	9.32 ± 3.42 E ijk	39.84 ± 4.08 C kl
A1	61.03 ± 1.97 C bc	81.40 ± 2.68 A bcd	74.25 ± 1.69 B abc	20.41 ± 1.07 E ef	55.67 ± 1.76 D fg
A2	56.29 ± 0.78 D de	76.98 ± 2.23 A defg	65.29 ± 1.28 B e	24.33 ± 1.22 E abc	61.97 ± 2.03 C e
A3	43.27 ± 1.15 D ij	73.32 ± 0.96 A h	58.04 ± 1.46 B fg	26.40 ± 1.02 E a	51.40 ± 0.96 C hi
A4	44.42 ± 1.26 D i	72.69 ± 0.86 A hi	56.72 ± 0.96 B fgh	23.52 ± 0.87 E bcd	49.65 ± 1.25 C hij

## Data Availability

The original contributions presented in this study are included in the article. Further inquiries can be directed to the corresponding author.
